# Identification of an Immune-Related Prognostic Predictor in Hepatocellular Carcinoma

**DOI:** 10.3389/fmolb.2020.567950

**Published:** 2020-09-24

**Authors:** Lei Wu, Wen Quan, Qiong Luo, Ying Pan, Dongxu Peng, Guihai Zhang

**Affiliations:** ^1^Department of Oncology, Zhuhai People’s Hospital (Zhuhai Hospital Affiliated With Jinan University), Zhuhai, China; ^2^Department of Oncology, Affiliated Zhuhai Hospital, Southern Medical University, Zhuhai, China

**Keywords:** hepatocellular carcinoma, PD-1, immune, HPD, PD-L1

## Abstract

Liver hepatocellular carcinoma (LIHC) is the most prevalent primary cancer of the liver, and immune-related genes (IRGs) regulate its development. So far, there is still no precise biomarker that predicts response to immunotherapy in LIHC. Therefore, this research seeks to identify immunogenic prognostic biomarkers and explore potential predictors for the efficacy of anti-PD-1/PD-L1 therapies in LIHC. The clinical data and gene expression profiles of patients diagnosed with LIHC were downloaded from The Cancer Genome Atlas (TCGA) and Gene Expression Omnibus (GEO) databases. Moreover, IRGs were obtained from the ImmPort database. We discovered 35 IRGs that were differentially expressed between LIHC tissues and corresponding normal tissues. Through univariate Cox regression analysis, eight prognostic differentially expressed IRGs (PDEIRGs) were identified. Further, three optimal PDEIRGs (BIRC5, LPA, and ROBO1) were identified and used to construct a prognostic risk signature of LIHC patients via multivariate Cox regression analysis. The signature was validated by ROC curves. Subsequently, based on gene set enrichment analysis (GSEA) analysis, two out of the three optimal PDEIRGs (BIRC5 and LPA) were significantly enriched in the mismatch repair (MMR) pathway. Moreover, the two PDEIRGs (BIRC5 and LPA) were significantly correlated with the expression of genes related to mismatch repair (MLH1, MSH2, MSH6, and PMS2). Furthermore, correlations between the two PDEIRGs (BIRC5 and LPA) and immune checkpoints of cancer treatment (such as CTLA4, PD-1, and PD-L1) were demonstrated. Hyperprogressive disease (HPD) is a novel pattern of tumor progression which has a close relationship with immune checkpoint inhibitors (ICIs) utilization. MDM2 family amplification might promote the HPD phenomenon. Finally, we found a positive regulatory relationship between HPD related gene (MDM2) and BIRC5. Notably, MDM2 can either interact directly with BIRC5 or indirectly via downstream transcription factors of BIRC5. Overall, our study uncovered a novel 3-immune-related prognostic genes in LIHC.

## Introduction

Liver cancer is the fourth most prevalent cause of cancer-related mortalities across the globe. The mortality rates have increased by 2.8% for males and 3.4% for females annually. Liver hepatocellular carcinoma (LIHC) accounts for over 90% of all cases of liver cancer for which immunotherapy and chemotherapy are the major approaches for therapy (Yang et al., 2019; Anwanwan et al., 2020). Therefore, predictive biomarkers of the prognostic and treatment of LIHC are urgently needed to improve the prognosis of LIHC patients.

In recent years, cancer immunotherapy based on checkpoint blockade has evolved as an emerging strategy for LIHC therapy. For instance, by blocking the PD-1/PD-L1 pathway, immune checkpoint inhibitors (ICIs) including, Pembrolizumab, Nivolumab, and Atezolizumab harbors important clinical applications with significantly favorable outcomes in LIHC (El-Khoueiry et al., 2017; Zhu et al., 2018; Hack et al., 2020). Nonetheless, the overall response rate of checkpoint inhibitors reaches only 15–20% in LIHC patients (Ma et al., 2019). As a consequence, there is an urgent need to identify sensitive biomarkers that predict ICIs response for LIHC.

Hyperprogressive disease (HPD) is a novel model of tumor progression, characterized by rapid progression in tumor volume. Several lines of evidence have reported that ICIs might induce an HPD, moreover, most of HPD cases have occurred in anti-PD-1/PD-L1 treatment, while a few in CTLA-4 treatment (Popat, 2019; Zang et al., 2020). For example, cancer patients with overexpression of MDM2 developed HPD after PD-1 inhibitor treatment (Kato et al., 2017). In addition, a previous study reported that MDM2 reduces activation of T cells by degrading transcription factor NFATc2, thereby causing resistance to PD-1 inhibitors of malignancies (Zou et al., 2014). Notably, the mechanism of MDM2 overexpression in the resistance to ICIs, particularly the HPD after immunotherapy of ICIs, needs further investigation.

Numerous research findings have demonstrated a relationship between IRGs and the response to immunotherapy, as well as the development and prognosis of LIHC patients. For instance, it has been found that LIHC specimens contain CD8 + T cells that express different levels of PD1, and LIHCs with a discrete population of PD1-high CD8 + T cells might be more susceptible to combined immune checkpoint blockade-based therapies (Kim et al., 2018). Also, CMTM7, as an IRG, was markedly downregulated in liver cancer tissues, and act as a tumor suppressor by blocking the progression of the cell cycle (Huang et al., 2019). ORM2 is a member of the immune family of genes, and a reliable prognostic factor for liver cancer (Zhu et al., 2020). Therefore, the IRGs based prognostic signature or biomarker for immunotherapy remains a potential to be applied in LIHC. Herein, based on IRGs, TCGA, GEO, and ImmPort databases were used to develop and verify a reliable prognostic signature for LIHC.

## Materials and Methods

### Data Collection

Hepatocellular carcinoma gene expression profiles of GSE62232, GSE84402, and GSE101685 were downloaded from the GEO^1^. The GSE62232 dataset comprised 91 samples including 81 LIHC tissues and 10 normal liver tissues. The GSE84402 dataset contained 28 samples including14 LIHC tissues and 14 normal liver tissues. On the other hand, the GSE101685 dataset contained 32 samples including 24 LIHC tissues and 8 normal liver tissues. Notably, GSE362232, GSE84402, and GSE101685 were all on account of the GPL570 platform. The clinical information and transcriptome expression profiles of LIHC were obtained from the TCGA database^2^. The TCGA dataset comprised 424 samples including 374 LIHC tissues and 50 non-tumorous tissues. A total of 1,811 IRGs were obtained from the ImmPort database^3^, which was funded by the NIH, NIAID, and DAIT in support of the NIH mission to share data with the public (Bhattacharya et al., 2018). Immune infiltrate data from the TCGA patients were obtained from the Tumor IMmune Estimation Resource (TIMER)^4^ (Li et al., 2016, 2017), a web server for comprehensive analysis of tumor-infiltrating immune cells.

### Identification of DEGs and DEIRGs

Using the limma package of R software, DEGs were identified by comparing LIHC tissues with normal liver tissues. Adjusted *P* < 0.05 and | log FC| > 1 were set as the cut-off values. Then, among the range of 1,811 IRGs, DEIRGs were screened out from the above DEGs.

### Development of the Immune-Related Signature for LIHC

Univariate analysis was used to identify immune-related genes with prognostic capability. Then, multivariate Cox proportional hazard regression was performed to select potential risk factors, and Cox proportional hazards regression was used to establish the prognostic immune signature. Further, the regression coefficients from the multivariate Cox regression signature were used to weigh the expression values of the selected genes. The formula of the risk score signature is described as follows: Risk score = (−0.0414 × LPA expression) + (0.02334 × BIRC5 expression) + (0.022119 × ROBO1 expression).

### Validation of Three PDEIRGs

The GEPIA database was used to analyze the expression of mRNA and protein levels of the three PDEIRGs (Tang et al., 2017) and the Human Protein Atlas database (HPA)^5^ (Pontén et al., 2011) respectively. Subsequently, the GEPIA database was used to perform survival predication.

### Human Cancer Transcription Factor Targets

The 318 cancer-related transcription factors (TFs) were obtained from the Cistrome Cancer Database^6^, which is a comprehensive resource for predicted TF targets and enhancer profiles in cancers (Liu et al., 2011).

### Gene Set Enrichment Analysis (GSEA)

GSEA was conducted to analyze the biological pathway in LIHC stratified by the median expression of BIRC5, LPA, and ROBO1. The detailed process followed the recommended protocol from the Broad Institute Gene Set Enrichment Analysis website (Subramanian et al., 2005). The GSEA was performed using the GSEA v4.0.3 software. NOM *p*-value at less than 0.05 (*p* < 0.05) and FDR *q*-value at less than 005 (*p* < 0.05) were considered statistically significant.

### PPI Network Construction and Module Analysis

Protein-protein interaction networks were constructed based on the STRING (Szklarczyk et al., 2019). The combined score >0.4 was selected for the construction of the PPI network. The PPI network was constructed with Cytoscape (version 3.6.0) software, and its modules screened using the MCODE app (version: 1.5.1).

### Statistical Analysis

All statistical analyses were performed using the R software (Version 3.6.1). For TCGA data, FPKM data pre-calculated by TCGA were used. Wilcox test was used to determine the differential gene expression between the tumor group and the normal group in the TCGA dataset. For GEO data, microarray expression data were normalized and analyzed using the R package “limma.” Hierarchical clustering heatmap illustrating the expression intensity of the DEGs was constructed using the pheatmap R package. Based on the median value of risk score, Kaplan-Meier curves were plotted and a log-rank test was used to check the significant difference in overall survival between high-risk and low-risk groups. Time-dependent receiver operating characteristic (ROC) analysis was used to evaluate the accuracy of the prognostic signature. An area under the ROC curve (AUC) acted as an indicator of prognostic accuracy. The AUC >0.60 was considered as acceptable for predictions (Han et al., 2018; Cho et al., 2019). Correlation coefficients were according to the method of Spearman. All tests were two-tailed paired *t*-test and *p*-values at less than 0.05 (*p* < 0.05) were considered statistically significant.

## Results

### Identification of DEGs in LIHC

**FIGURE 1:**
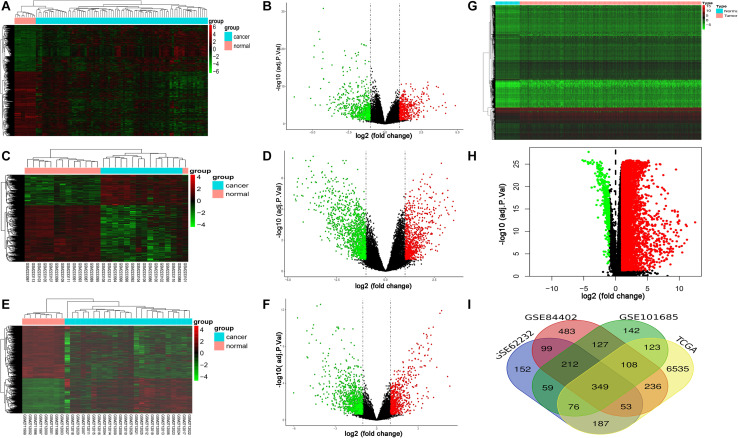
Expression of DEGs between LIHC and normal liver tissues in GSE62232, GSE84402, GSE101685, and TCGA datasets. **(A,B)** Heat map and volcano plot of DEGs from GSE62232 dataset. **(C,D)** Heat map and volcano plot of DEGs from GSE84402 dataset. **(E,F)** Heat map and volcano plot of DEGs from GSE101685 dataset. **(G,H)** Heat map and volcano plot of DEGs from TCGA dataset. **(I)** Authentication of 349 common DEGs in the four datasets (GSE62232, GSE84402, GSE101685, and TCGA) through Venn diagrams software.

GSE62232, GSE84402, and GSE101685 datasets were obtained from the GEO. Microarray data of GSE362232, GSE84402, and GSE101685 were all based on the platform of GPL570 which included 81 LIHC tissues and 10 normal liver tissues, 14 LIHC tissues and 14 normal liver tissues, 24 LIHC tissues and 8 normal liver tissues, respectively. The TCGA dataset contained 424 samples including 374 LIHC tissues and 50 non-tumorous tissues. Considered as the criteria of adjust | log FC| > 1 and *P* < 0.05. A total of 1,187, 1,667, 1,196, and 7,667 DEGs were detected from GSE362232, GSE84402, GSE101685, and TCGA, respectively (Figures 1A–H). In total, 349 commonly DEGs were identified through the comprehensive analysis of four datasets (Figure 1I).

### Identification of DEIRGs in LIHC

**FIGURE 2:**
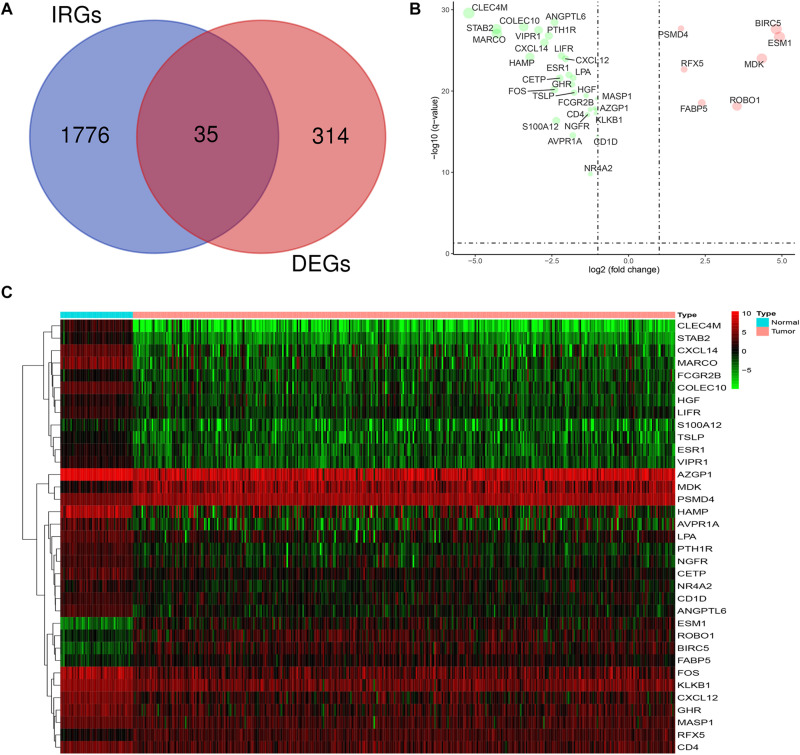
Expression of DEIRGs in the DEGs of LIHC. **(A)** Authentication of 35 DEIRGs among the 349 DEGs through Venn diagrams software. **(B)** The volcano plot of DEIRGs; red dots indicate up-regulated DEIRGs, while green dots indicate down-regulated DEIRGs. **(C)** Heat map of DEIRGs.

The mRNA expression levels of 1,811 IRGs were examined among the aforementioned 349 DEGs of LIHC. The analysis revealed 35 differentially expressed IRGs (DEIRGs), including 7 upregulated and 28 downregulated genes in LIHC tissues compared to normal liver tissues (Figure 2). Considered as the criteria of adjust *P* < 0.05 and | log FC| > 1.

### Construction of an Immune-Related Prognosis Signature for LIHC

**FIGURE 3:**
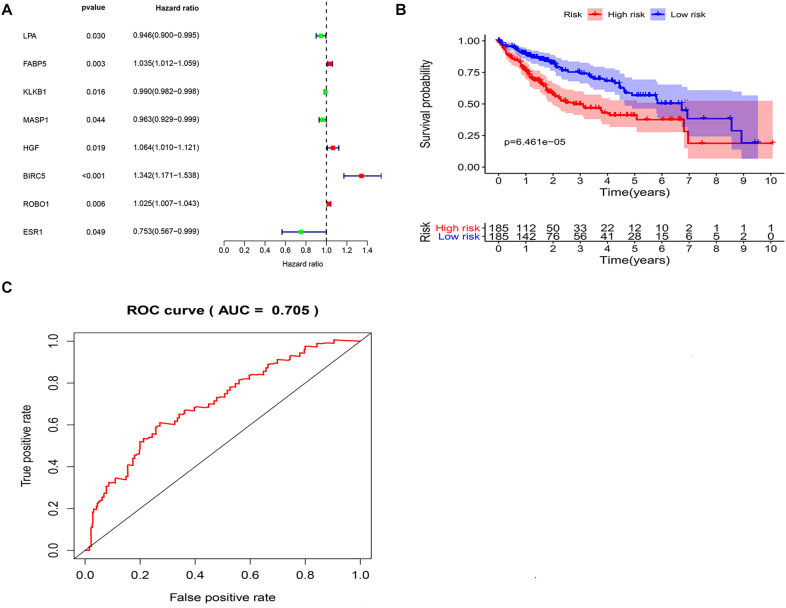
Identification of potential immune-related prognostic signature for LIHC. **(A)** Forest plot of the relationship between DEIRGs and OS of patients with LIHC. **(B)** KM curves of high and low risk score groups. **(C)** ROC curve of the 3-gene signature prognostic signature.

To explore potential prognostic DEIRGs (PDEIRGs) in LIHC, the univariate Cox regression analysis was performed to investigate the prognostic value of 35 DEIRGs in 374 patients with LIHC in the TCGA. In total, eight PDEIRGs showed a significant correlation with the OS of patients with LIHC (*p* < 0.05). Among the eight genes, four genes including FABP5, HGF, BIRC5, and ROBO1 were identified as high-risk factors (*p* < 0.05, *HR* > 1) and four genes among them, LPA, KLKB1, MASP1, and ESR1 were identified as low-risk factors (*p* < 0.05, *HR* < 1) (Figure 3A). Then, a multivariate Cox regression was used to develop the following immune-related risk signature associated with the survival of LIHC patients. The formula for risk score was as follows: Risk score = (−0.0414 × LPA expression) + (0.02334 × BIRC5 expression) + (0.022119 × ROBO1 expression). Subsequently, using the median risk score as a cutoff value, the patients were divided into the low-risk group (*n* = 185) and high-risk group (*n* = 185). Our data showed that the survival time of the high risk group was significantly shorter than the low-risk group (Figure 3B). ROC curve was generated to assess the prognostic accuracy of the signature for OS at 1 year, the AUC of the signature was 0.705 (Figure 3C).

### Independent Prognostic Value of the Risk Signature in the LIHC

**FIGURE 4:**
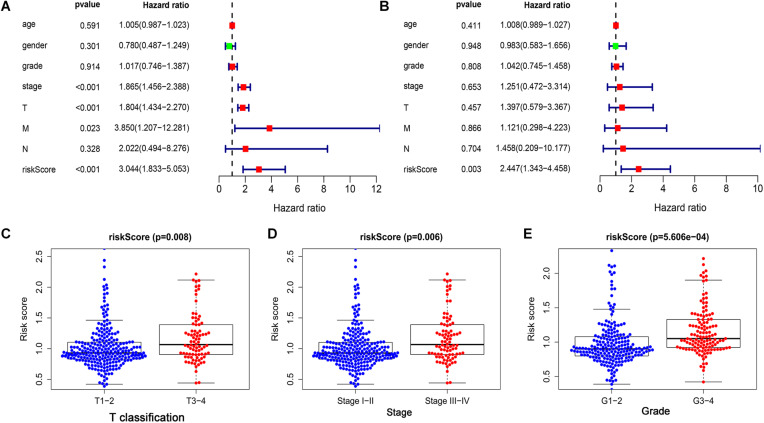
The prognostic value of the risk score was assessed in LIHC. **(A)** The correlation between clinical parameters and risk score were assessed through a univariate Cox regression and **(B)** multivariate Cox-regression. **(C)** The correlation between risk score and T classification. **(D)** The correlation between risk score and clinical stage. **(E)** The correlation between risk score and histological grade.

Univariate and multivariate Cox regression analyses were performed to further examine the independence of the risk score to other clinical parameters including, age, gender, histological grade, clinical stage, T stage, N stage, and M stage as a prognostic factor for LIHC. Univariate analysis indicated that the variables of clinical-stage, T stage, M stage, and risk score were significantly correlated with the prognosis of LIHC patients (*p* < 0.05) (Figure 4A). In multivariate analysis of clinical parameters, the forest plot showed that the risk score was an independent factor correlated with OS in the LIHC patients (Figure 4B). Further, the association between the above clinical parameters and risk score was investigated, and the results indicated that T classification, clinical stage and histological grade were associated with the risk score (all *p* < 0.05) (Figures 4C–E). Therefore, these findings suggested that the prognostic risk signature could act as an independent factor in predicting the prognosis of LIHC patients.

### Identification of the Relationship Between the Immune Cell Infiltration and Risk Score in LIHC Patients

**FIGURE 5:**
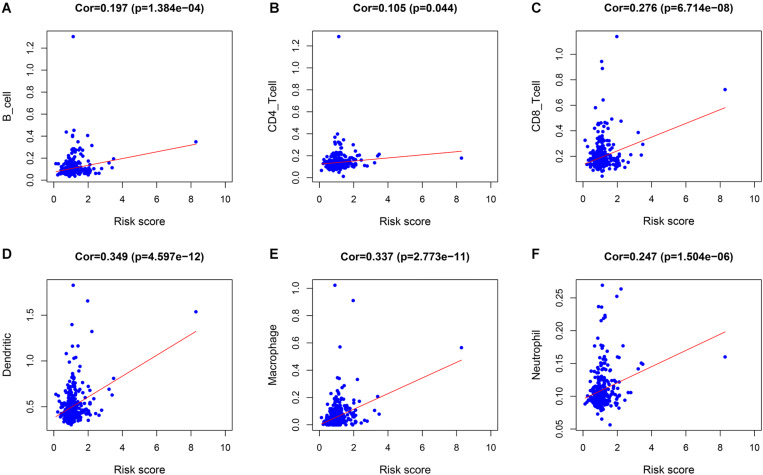
The relationship between the immune cell infiltration and risk score in LIHC patients. **(A)** B cells. **(B)** CD4 + T cells. **(C)** CD8 + T cells. **(D)** Dendritic cells. **(E)** Macrophages. **(F)** Neutrophils.

The correlations between the risk score and the infiltration of six immune cell types in LIHC were estimated to examine whether our signature could reflect the status of the tumor immune microenvironment in patients. As the level of risk score increased, the six types of immune cells including B cells, CD4+T cells, CD8+T cells, dendritic cells, macrophages, and neutrophils in LIHC tissues were also increased (Figures 5A–F). In further immune cell subtype refinement, as the level of risk score increased, the five types of immune cells including T cells CD4 memory activated, T cells follicular helper, T cells gamma delta, B cells memory, and Macrophages M0 in LIHC tissues were also increased (Supplementary Figures 1A–F).

### Protein and mRNA Expression Levels of the Three PDEIRGs Among the Risk Signature in LIHC

**FIGURE 6:**
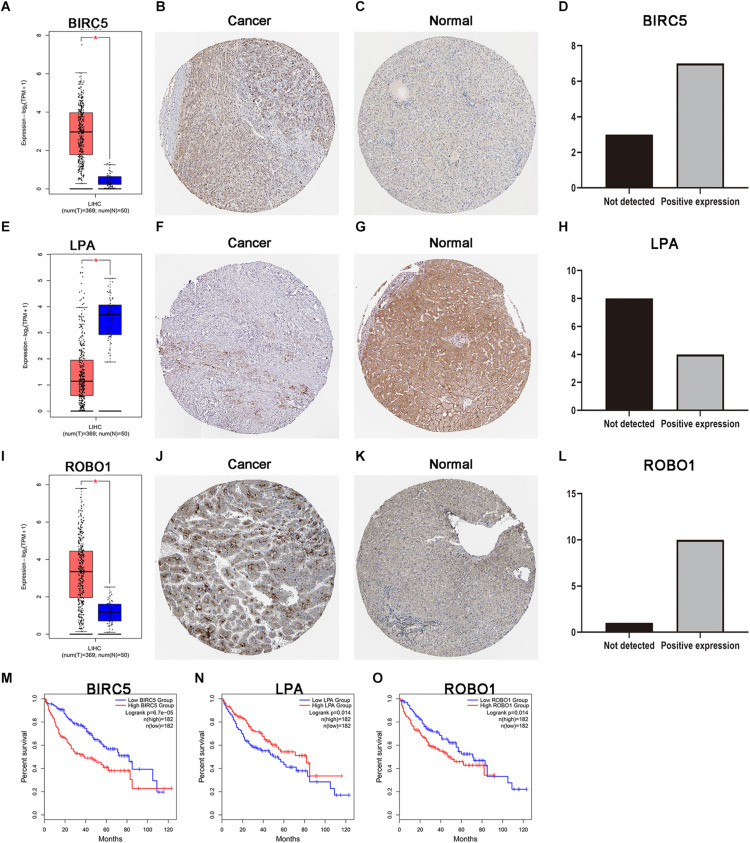
Expression and survival curve of BIRC5, LPA, and ROBO1. **(A,E,I)** BIRC5, LPA, and ROBO1 expression in normal and malignant liver tissue sampled from 419 patients using GEPIA. **(B–D)** Immunohistochemistry (IHC) staining for BIRC5 in tumor liver tissue and normal liver tissue from a Human Protein Atlas Portal. **(F–H)** IHC staining for LPA in tumor liver tissue and normal liver tissue from a Human Protein Atlas Portal. **(J–L)** IHC staining for ROBO1 in tumor liver tissue and normal liver tissue from a Human Protein Atlas Portal. **(M–O)** Kaplan–Meier survival curves of BIRC5, LPA, and ROBO1 in LIHC patients, respectively. *P* < 0.05 was as statistically significant.

As discussed above, BIRC5, LPA, and ROBO1 significantly predicted survival in LIHC patients. Further, the expression of three PDEIRGs at the mRNA level and protein level in LIHC patients were analyzed using the available data in the GEPIA database and Human Protein Atlas. Results demonstrated high mRNA expression of BIRC5 and ROBO1 in LIHC samples compared to normal liver samples (Figures 6A,I). Meanwhile, results revealed low mRNA expression of LPA in LIHC samples compared to normal liver samples (Figure 6E). In addition, IHC results showed that normal liver tissue stained negatively or weakly positive for BIRC5 and ROBO1, while tumor tissue was high or medium positive (Figures 6B,C,J,K). The expression of BIRC5 was detected in 3/10 cases (30%), while positive expression of BIRC5 was found in 7/10 cases (70%) of liver cancer tissues (Figure 6D). Additionally, the expression of ROBO1 was detected in 1/11 cases (9%), while positive expression of ROBO1 was found in 10/11 cases (91%) of liver cancer tissues (Figure 6L). However, at the protein expression level of LPA, normal liver tissue stained positive, while tumor tissue was negative or weakly positive (Figures 6F,G). The expression of LPA was detected in 8/12 cases (67%), while high and medium LPA was detected in 4/12 cases (33%) of liver cancer tissues (Figure 6H). Then, the K-M analysis of the above three PDEIRGs (BIRC5, LPA, and ROBO1) was performed using the GEPIA database. A high expression of BIRC5 and ROBO1 was related to a worse OS in LIHC patients (Figures 6M,O), while high expression of LPA predicted effective prognosis of LIHC patients (Figure 6N).

### Regulatory Network of the Transcription Factors-PDEIRGs

**FIGURE 7:**
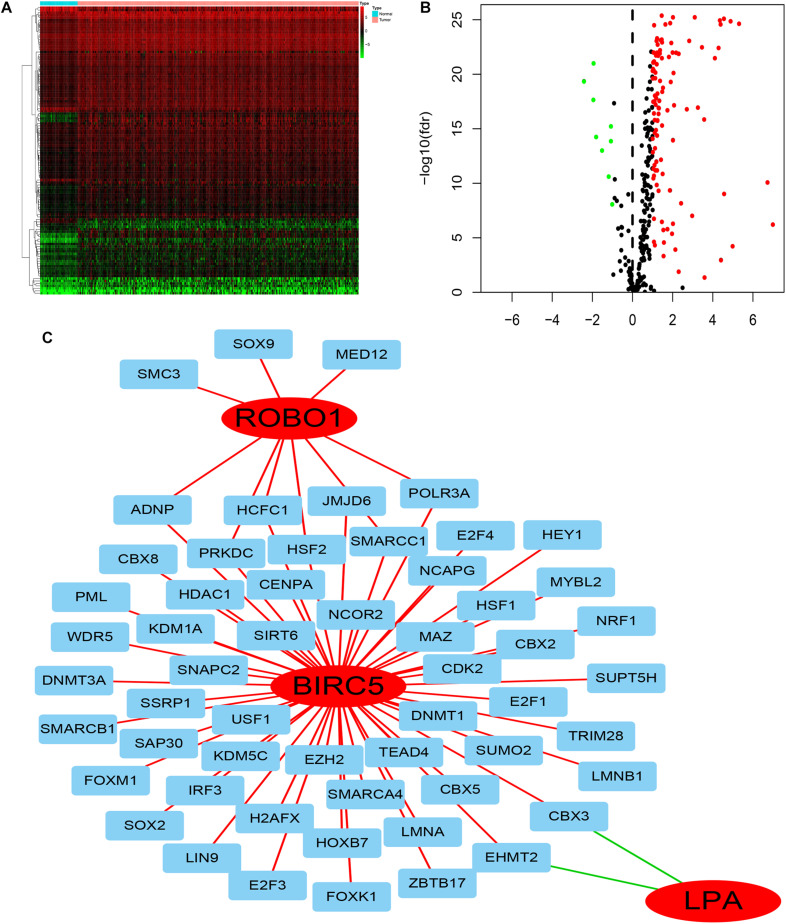
The regulatory network of TF-PDEIRGs. **(A,B)** Heat map and Volcano plot of differentially expressed TFs. **(C)** Regulatory network of three PDEIRGs and TFs; red lines indicate positive regulatory correlations, while green lines suggest negative regulatory correlations. The red nodes represent PDEIRGs, the blue nodes represent TFs that correlated with the PDEIRGs.

To deduce the possible mechanisms behind the dysregulation of the three PDEIRGs among the risk signature in LIHC, the relationship between the three PDEIRGs and cancer TFs expression was investigated. First, the mRNA expression levels of TFs in LIHC (*n* = 374) and normal liver tissues (*n* = 50) were analyzed, where a total of 117 differentially expressed TFs were identified between the two tissue types (FDR < 0.05, | log FC| > 1) (Figures 7A,B). Then, the correlation between 117 TFs expression and three PDEIRGs expression at the mRNA level was analyzed, *p* < 0.05 was used as the threshold. Among the 117 TFs, 64 TFs were significantly associated with the aberrant expression of three PDEIRGs. Further, a regulatory network was constructed to effectively investigate the regulatory associations (Figure 7C).

### GSEA Analysis of Three PDEIRGs in LIHC

**FIGURE 8:**
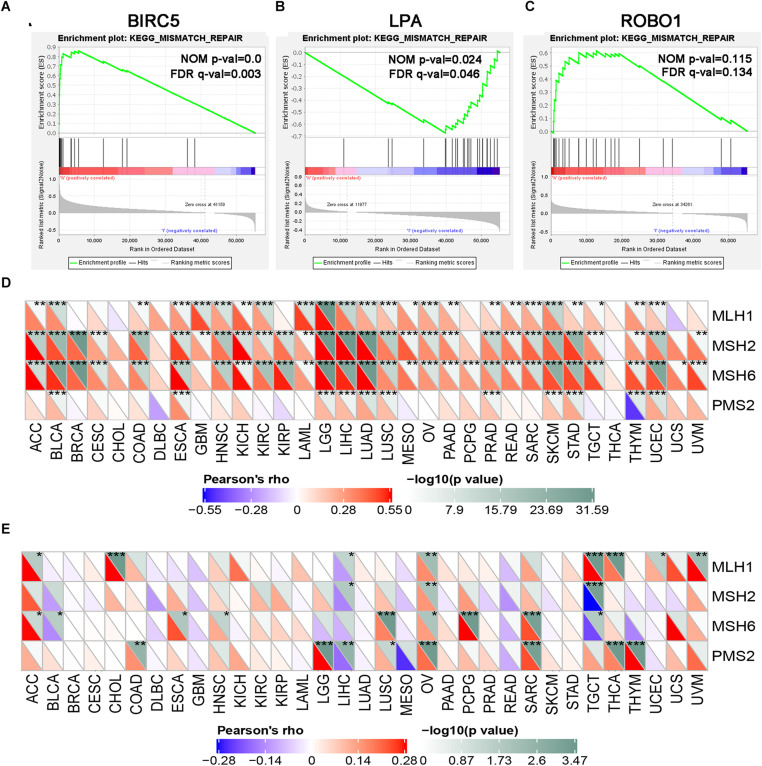
GSEA enrichment analysis of three PDEIRGs in LIHC. Individual gene set enrichment plots of GSEA results by **(A)** BIRC5 expression, **(B)** LPA expression, **(C)** ROBO1 expression. **(D)** The correlation between BIRC5 and four major genes of the mismatch repair. **(E)** The correlation between LPA and four major genes of the mismatch repair. *Represents *P* < 0.05, **represents *P* < 0.01, ***represents *P* < 0.001.

GSEA was performed for each gene to determine the immunotherapy mechanism of BIRC5, LPA, and ROBO1. The GSEA results revealed that all of the three genes were enriched in mismatch repair. However, the result was not statistically significant in the ROBO1 group (Figures 8A–C). Further, we analyzed the correlation between BIRC5 and LPA and four major genes among them, MLH1, MSH2, MSH6, and PMS2 of the mismatch repair was analyzed. As a result, a positive relationship was detected between BIRC5 and four major genes of the mismatch repair at mRNA expression levels (Figure 8D). For the LPA group, the expression of LPA was negatively regulated with the expression of MLH1, MSH2, and PMS2 (Figure 8E).

### The Correlation of Immune Checkpoint Molecules Expression With BIRC5 and LPA in Pan-Cancer

**FIGURE 9:**
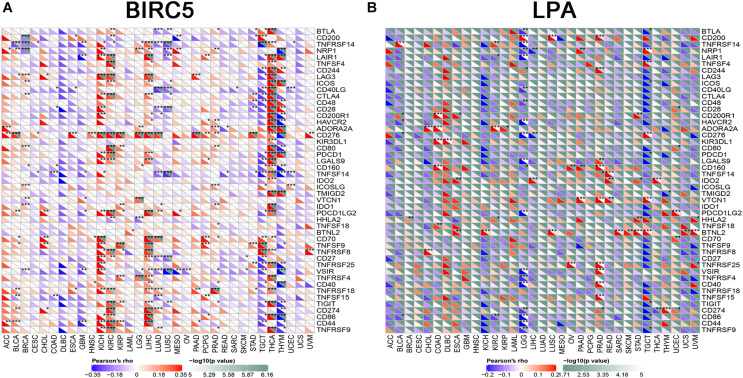
The correlation of expression of Immune Checkpoint Molecules with **(A)** BIRC5 and **(B)** LPA in Pan-Cancer. The abscissa is different types of tumors; ordinate is different immune checkpoints. The red triangle represents positive correlations. The blue triangle represents negative correlations. The gray triangles represents. *P*-value. *Represents *P* < 0.05, **represents *P* < 0.01, ***represents *P* < 0.001.

To further test the correlation of BIRC5 and LPA with immunotherapy, the expression association of known immune checkpoint genes (Danilova et al., 2019) with BIRC5 and LPA was analyzed. As shown in Figure 9A, a positive correlation between the mRNA expression level of BIRC5 and PDCD1 (PD-1), CD274 (PD-L1) was observed respectively in LIHC. However, there was no correlation observed between LPA and PDCD1 (PD-1), CD274 (PD-L1), respectively, in LIHC (Figure 9B).

### Hyperprogressive Disease-Related Gene and BIRC5

**FIGURE 10:**
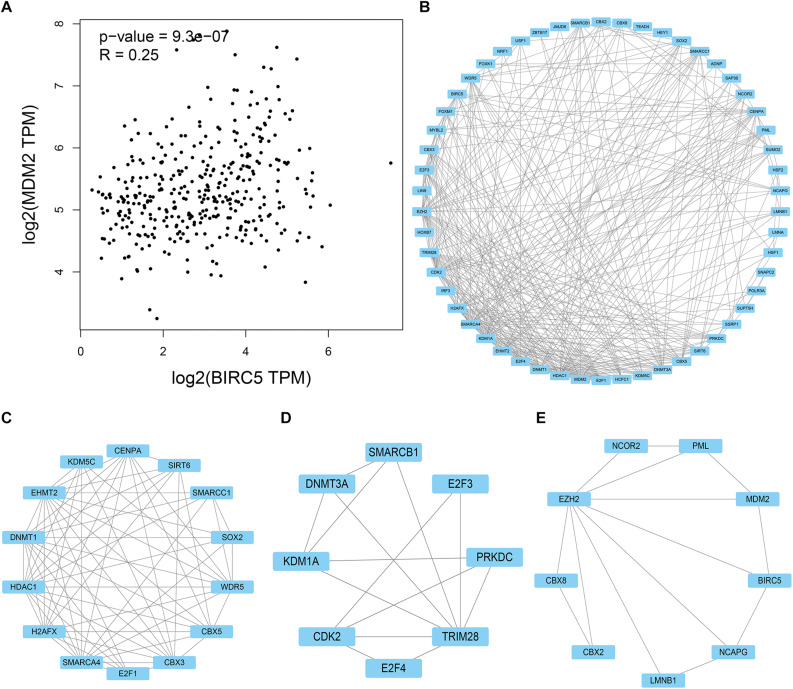
PPI network of BIRC5 and its associated tumor transcription factors and MDM2 was analyzed by Cytoscape software. **(A)** The association between mRNA expression levels of BIRC5 and MDM2 in LIHC. **(B)** Cluster of the total central PPI network. **(C–E)** The top three clusters from the PPI network.

HPD is a novel model of progression, with rapid progression in tumor volume. Past research reported that ICIs might induce an HPD, and MDM2 family amplification might participate in HPD phenomenon (Kato et al., 2017; Popat, 2019). As shown in Figure 10A, mRNA expression levels of BIRC5 from patient samples obtained from the TCGA database were positively associated with MDM2. STRING and Cytoscape were used to determine whether the BIRC5 and its associated tumor transcription factors predicted before were functionally related to MDM2 (Figure 10B). An MCODE plug-in was used in Cytoscape to build a PPI network with 54 nodes and 331 edges, the top 3 hub clusters with the highest node degrees were shown (Figures 10C–E). Notably, MDM2 can either interact directly with BIRC5 or indirectly via downstream transcription factors of BIRC5 (Figure 10E).

## Discussion

Accumulating evidence has demonstrated that immune cells regulate the occurrence and development of multiple tumors (Grivennikov et al., 2010; Wu et al., 2015). Notably, immune cells can kill tumor cells by regulating IRGs at certain immune checkpoints (Rooney et al., 2015; Sayour and Mitchell, 2017). Nevertheless, cancer cells can regulate the IRG expression patterns of healthy cells thereby dampening antitumor immune responses (Sharma et al., 2017; Friedrich et al., 2019). A previous study found that hepatocellular carcinoma progression is accompanied by immune resistance and immune evasion (Zhang et al., 2017). Therefore, IRG might be a significant marker in predicting the prognosis and progression of LIHC. Here, we found eight prognosis-associated IRGs, and three of them were used to build a reliable signature which predict the prognosis of LIHC patients.

First, we identified 35 DEIRGs from 1,811 IRGs in LIHC, including 7 up-regulated and 28 down-regulated genes. Then, a correlation of eight DEIRGs with the OS of LIHC patients was demonstrated via univariate Cox regression analysis. Through a multivariate regression analysis, we identified three PDEIRGs (BIRC5, LPA, and ROBO1), and constructed a Cox regression hazard signature. The signature was identified as an independent prognostic factor of LIHC patients in the Cox regression model analysis. Therefore, our signature might improve the prognosis of patients diagnosed with LIHC in clinical practice.

A TF regulatory network was constructed to explore the potential molecular mechanisms underlying the role of the three PDEIRGs (BIRC5, LPA, and ROBO1) in LIHC. As a result, a total of 64 TFs were found to be correlated with the three PDEIRGs. These findings demonstrated that TFs determined the impact of the PDEIRGs on the overall survival of patients with LIHC.

Of note, the degree of immune cell infiltration exhibits a severe influence on the prognosis of LIHC. Chew et al. (2012) discovered that the infiltration of tumors by NK and T cells has been linked to survival in LIHC. Subsequently, investigators further identified that gene sets for CD8+ cells, NK cells, macrophages, immature dendritic cells, and T cell co-stimulation were associated with survival in LIHC (Foerster et al., 2018). Thus, this study also investigated the relationship between the infiltration of six immune cells and the risk score from our signature. We found that the six types of immune cells were positively associated with the risk score.

Cancer immunotherapies trigger antitumor effects by inducing or enhancing immune responses of patients (Tsimberidou et al., 2018). Numerous investigators have confirmed that ICIs are a broadly effective class of immunotherapies that reactivate immune responses against cancer (Cohen et al., 2019; Kim et al., 2019). In recent decades, a number of studies showed promising results in the application of ICIs in LIHC (El Dika et al., 2019). For instance, by blocking the PD-1/PD-L1 pathway, ICIs such as Nivolumab, Pembrolizumab, and Atezolizumab exhibit crucial clinical applications with significantly favorable outcomes in LIHC (El-Khoueiry et al., 2017; Zhu et al., 2018; Hack et al., 2020). Nonetheless, the overall response rate of ICIs is only approximately 15–20% in LIHC patients (Ma et al., 2019). As a consequence, there is an urgent need to identify effective prognostic biomarkers for LIHC patients who can benefit or fail to benefit from ICIs treatment.

DNA mismatch repair (MMR) is an essential pathway where there is a coordinated function of multiple protein complexes in repair of DNA damage (Iyer et al., 2006). MLH1, MSH2, MSH6, and PMS2 are the primary proteins implicated in the MMR system (Li, 2008). DNA MMR-deficient (dMMR) causes an increased rate of mismatch errors, which further triggers microsatellite instability (MSI) (Lynch et al., 2010). Notably, DNA MMR-deficient phenomenon is common in most types of tumors (Ratner and Lennerz, 2018). A previous study found that 42 out of 149 tumor specimens exhibited loss of MMR protein by IHC (Yamashita et al., 2018). Moreover, MSI and dMMR were identified as predictive biomarkers that guide the clinical application of ICIs therapies (Yi et al., 2018).

Based on GSEA analysis, we found that BIRC5 was significantly enriched in the MMR pathway. Moreover, BIRC5 significantly and positively correlated with the expression at the transcriptome level of main genes related to MMR (MLH1, MSH2, MSH6, and PMS2). Therefore, this implied that BIRC5 levels were elevated in LIHC compared to normal tissues. Highly expressed BIRC5 positively regulates the expression of the four key genes in MMR thus might increase the stability of the MMR system. Under these circumstances, the efficacy of anti-PD1/PD-L1 therapies might be less sensitive in LIHC.

In tumor immunotherapy, a new model of progression, with rapid tumor progression induced by anti-PD-1/PD-L1 treatment known as hyper-progressive disease (HPD) was observed (Champiat et al., 2017). Elsewhere, a study found that advanced cancer patients with MDM2 overexpression developed HPD after treatment with PD-1 inhibitor (Kato et al., 2017). Meanwhile, given that MDM2 acts as a tumor-associated antigen, immunological tolerance might also promote HPD induced by MDM2 overexpression (Bendle et al., 2005; Mayr et al., 2006). MDM2 is overexpressed in numerous cancer cell lines and binds on p53, causing the escape of cancer cells from p53-regulated control (Oliner et al., 1992). Overexpression of the BIRC5 gene in breast cancer cells upregulates the levels of MDM2 and downregulates the expression of the p53 gene thereby inhibiting the apoptotic effect induced by the p53 pathway (Wang et al., 2004). Similarly, a previous study found that selective inhibition of histone deacetylase 2 (HDAC2) causes downregulation of BIRC5 through activation of p53, which is mediated by the downregulation of MDM2 in lung cancer (Seo et al., 2015). The above studies have confirmed an apparent relationship between MDM2 and BIRC5.

Furthermore, we discovered that the mRNA expression level of MDM2 in LIHC was positively correlated with BIRC5. Further analyses by STRING and Cytoscape found that MDM2 can either interact directly with BIRC5 or indirectly via downstream transcription factors of BIRC5. Therefore, we hypothesized that through its special association with MDM2, over-expressed BIRC5 reduced the sensitivity of anti-PD1/PD-L1 therapy in LIHC.

## Conclusion

In conclusion, a reliable three immune-related gene signature was constructed and validated geared toward precisely predicting the prognosis of patients diagnosed with LIHC. We found that the risk score contributes to new independent clinical biomarkers of LIHC. However, in-depth investigations and prospective studies are essential to validate our findings.

## Data Availability Statement

Publicly available datasets were analyzed in this study. This data can be found here: The Cancer Genome Atlas (https://portal.gdc.cancer.gov/) and the NCBI Gene Expression Omnibus.

## Author Contributions

LW and GZ conceived and designed the study. WQ and QL analyzed the data. YP and DP revised the images. LW drafted the manuscript. GZ revised the manuscript. All authors contributed to the article and approved the submitted version.

## Conflict of Interest

The authors declare that the research was conducted in the absence of any commercial or financial relationships that could be construed as a potential conflict of interest.

## Footnotes

1 https://www.ncbi.nlm.nih.gov/geo/

2 https://portal.gdc.cancer.gov/

3 https://www.immport.org/home

4 https://cistrome.shinyapps.io/timer/

5 http://www.proteinatlas.org/

6 http://cistrome.org/
