# Supplementary material for: Identification of an Immune-Related Prognostic Predictor in Hepatocellular Carcinoma
Source: Front Mol Biosci. 2020 Sep 24;7:567950. doi: 10.3389/fmolb.2020.567950 (PMC7542239; doi:10.3389/fmolb.2020.567950)
Supplement: Supplementary file 2 [file Data_Sheet_2.PDF]

Supplementary Figure 1:

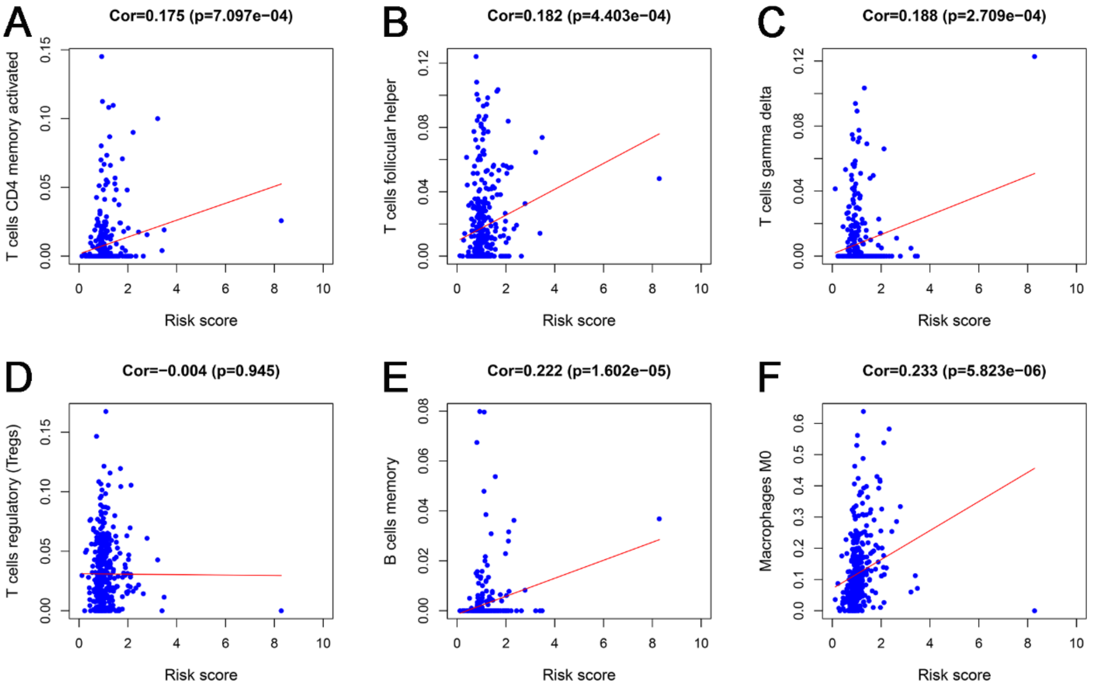

The relationship between the immune cell infiltration and risk score in LIHC patients. (A) T cells CD4 memory activated (B) T cells follicular helper (C) T cells gamma delta (D) T cells regulatory (Tregs) (E) B cells memory (F) Macrophages M0.
